# WHAT COMES FIRST? SENTINEL CHRONIC AND SUBSEQUENT MULTIMORBIDITY AMONG OLDER ADULTS

**DOI:** 10.1093/geroni/igae098.2408

**Published:** 2024-12-31

**Authors:** Dongmei Zuo, Solveig Cunningham

**Affiliations:** Emory University, Atlanta, Georgia, United States; Emory University, Atlanta, Georgia, United States

## Abstract

The majority of the U.S. adult population is affected by at least one chronic condition. To develop clinical practice guidelines and early prevention interventions, it is necessary to understand the progression of multimorbidity. We investigated the first chronic condition, or sentinel condition, developed by individuals using 15 waves of longitudinal data from the Health and Retirement Study, 1992-2020. Hypertension, arthritis, and heart diseases emerged as the most frequent sentinel conditions. Employing weighted multinomial logistic regression models, we assessed the association of onset age, sex, and race/ethnicity with the likelihood of these conditions relative to a combined category of cancer, diabetes, lung diseases, or stroke as the first onset disease, adjusting for education, marital status, and household income. We find that, compared to having cancer, diabetes, lung, or stroke diseases as the sole first onset condition, each additional year of age decreases the odds of having hypertension and heart disease as the first onset disease by 1.9% and 2.2%, respectively, and decreases the odds of arthritis by 1.8%. Being Hispanic and non-Hispanic Black increases the odds of hypertension as the first onset disease by 64% and 19%, respectively. Being female increases the odds of arthritis by 60%, while being Hispanic increases the odds by 16%, and being non-Hispanic Black decreases the odds by 44%. For multimorbidity, being female and non-Hispanic Black decreases the odds by 12% and 36%, respectively, while being Hispanic increases the odds by 55%, relative to cancer, diabetes, lung diseases, or stroke as the first onset disease.

